# Mahaim Fiber Accelerated Automaticity and Clues to a Mahaim Fiber Being Morphologically an Ectopic or a Split AV Node

**Published:** 2010-01-07

**Authors:** Shomu Bohora, Narayanan Namboodiri, Santosh Dora, VK Ajit Kumar, Jaganmohan Tharakan

**Affiliations:** Sree Chitra Tirunal Institute for Medical Sciences and Technology, Trivandrum, Kerala, India-695011.

**Keywords:** Mahaim fiber, automaticity, tachycardia

## Abstract

Mahaim Fiber tachycardia characteristically causes a wide QRS tachycardia with left bundle branch morphology and left axis deviation, especially in young patients, having no structural heart disease. Mahaim fiber automaticity further cements the proposition of Mahaim fiber, due to its Atrioventricular (AV) node like property, being called as an ectopic AV node.

## Case Report

**Figure 1:**
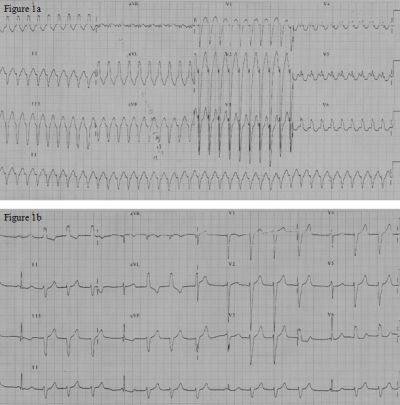
a:  12 lead Electrocardiogram showing a broad QRS tachycardia with Axis of -60 degrees, LBBB morphology and initial sharp r in V1 suggesting a Mahaim fiber tachycardia. b: 12 lead Electrocardiogram showing the presence of Mahaim automatic rhythm interspersed with Hisian escape beats also. The Mahaim automatic rhythm is more than the Hisian escape rate and is nearly at a rate of 100/min. The sinus node is suppressed immediately after tachycardia.

**Figure 2:**
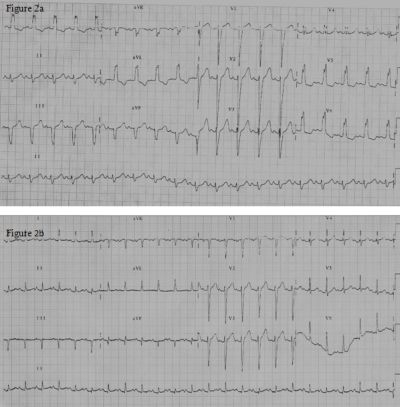
a: 12 lead electrocardiogram of the patient in sinus rhythm and normal PR interval having the same QRS morphology as the tachycardia and the automatic rhythm suggesting preferential antegrade conduction via the Mahaim fiber. b: 12 lead electrocardiogram showing narrow QRS in sinus tachycardia.

**Figure 3:**
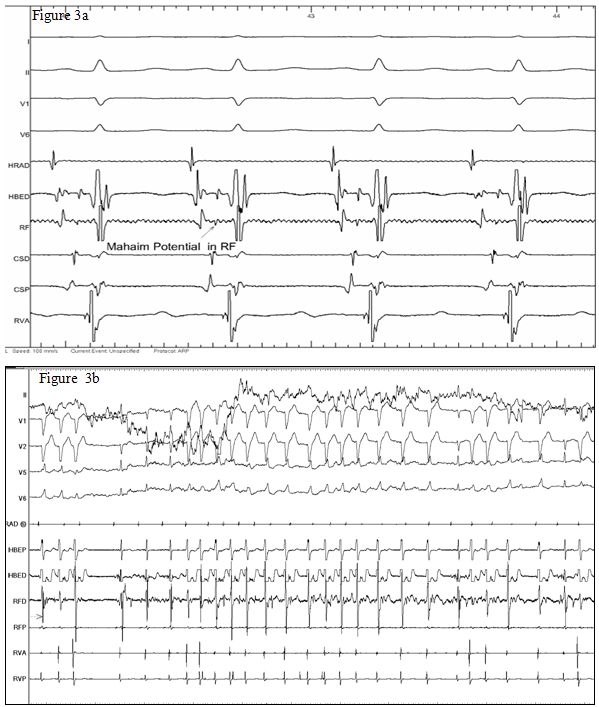
a: EP tracing showing the Mahaim fiber potential in the RF channel (marked by arrow), which is nearly similar to the potentials recorded in the His catheter (HBED channel) (Paper speed 100 mm/sec). b: Mahaim accelerated rhythm during RF ablation.

A 12-year-old girl presented with history of paroxysmal palpitations. The tachycardia electrocardiogram (ECG), as shown in Figure 1a, shows a broad QRS tachycardia (heart rate 210/min) with left bundle branch (LBBB) and left axis deviation (LAD) (QRS axis of -60 degree). She had no structural heart disease and had good left ventricular function on echocardiogram. Adenosine (6 mg) given intravenously terminated the tachycardia. However the tachycardia immediately reappeared. Since adenosine had once terminated the tachycardia, intravenous Verapamil 2.5 mg was given. The tachycardia terminated. The rhythm after tachycardia termination as shown in Figure 1b, showed an escape rhythm, which was predominantly a wide QRS escape, morphology of which was similar to the tachycardia, interspersed with narrow QRS escape beats. P waves were not well delineated. This wide QRS escape rate was nearly at 100 beats per minute. Within two minutes, sinus rhythm with a wide QRS morphology was noted; again similar to the tachycardia QRS morphology, with a normal PR interval (Figure 2a). A narrow QRS morphology in sinus rhythm, which persisted later on, was noted thereafter (Figure 2b). The patient was suspected to have a Mahaim fiber tachycardia (atrio-fascicular tachycardia) related symptoms. During the electrophysiology (EP) study, incremental atrial stimulation showed gradual shortening of the HV interval with the appearance of ventricular pre-excitation, with right bundle getting activated earlier than the His. There was decremental conduction through the accessory pathway. Atrial pacing initiated the tachycardia, which, showed that right bundle branch (RBB) was activated prior to the His bundle during the tachycardia. The antegrade refractory period of both the Atrioventricular (AV) Node and the Mahaim fiber was 290 msec. Ventricular stimulation showed earliest atrial activity at the His bundle region due to retrograde conduction via the AV node.  An SR 0 sheath was positioned in the right atrium via the right femoral vein, and a 7F RF catheter (BARD Stinger, D-curve) was passed via the sheath and the lateral tricuspid annulus was mapped. Mahaim fiber potential was recorded at the 8 o'clock position at the tricuspid annulus (Figure 3a), where radio frequency ablation (RFA) was successful. Mahaim junctional accelerated rhythm was noted during the initially during the ablation (Figure 3b) which was continued for 90 seconds. There was no further conduction via the accessory pathway and tachycardia was not inducible by stimulation protocols. Patient has remained asymptomatic after RFA.

## Discussion

Mahaim fiber tachycardia should be considered as a differential diagnosis for a wide QRS tachycardia with LBBB morphology and LAD, in a patient having no structural heart disease. The sequence of events in this patient gives clues for the presence of Mahaim fiber pathway.

Initially tachycardia had a broad QRS, LBBB morphology and left axis deviation, with an initial sharp R in lead V1, which is characteristic of Mahaim fiber tachycardia. Thereafter with termination of the tachycardia, a Mahaim fiber escape rhythm, which was accelerated, interspersed with occasional Hisian escape, which suggests higher automaticity of the Mahaim fiber in comparison with the AV node, was noted. Mahaim fiber spontaneously automaticity has been very infrequently observed and documented and most reports have been when the patient was undergoing a Holter test [1,2] or an EP study [1,3,4]. Mahaim automaticity and competing rhythm with AV node escape rates of 65-72/min have been described earlier [3].

Wide QRS morphology similar to the tachycardia, during sinus rhythm, with a normal PR interval, is suggestive of preferential antegrade conduction through the Mahaim fiber tract, and narrow QRS morphology during sinus rhythm, suggest antegrade conduction through the AV node, which is because of the near similar antegrade refractory periods of the Mahaim pathway and the AV node. The presence of a Mahaim fiber potential (M Potential), is similar to an intra-cardiac electrogram of a His bundle potential, suggests the optimal site of ablation. During ablation Mahaim fiber accelerated rhythm has been considered a marker of successful ablation, which is akin to the junctional accelerated rhythm during slow pathway ablation. All these properties of Mahaim fiber point towards it being akin to AV Node. This case report highlights the presence of nearly all features of Mahaim fiber behaving as an ectopic AV node.

## Conclusion

Mahaim fiber shows characteristics of a decremental conducting antegrade pathway, on an anatomical location, usually, just diagonally opposite the AV node on the tricuspid annulus. Insertion point of the Mahaim tract is into the right bundle in most instances, with near similar antegrade refractory periods as the His bundle. The intra-cardiac electrogram of Mahaim fiber is similar to a His bundle electrogram. Mahaim fiber also demonstrates automatic/escape rhythm and an accelerated rhythm during ablation. Thus Mahaim fibers share common features as the His conduction system and hence probably Mahaim fiber tract, represents developmentally as an ectopic AV node or a split AV node.
